# NETs-related genes predict prognosis and are correlated with the immune microenvironment in osteosarcoma

**DOI:** 10.3389/fonc.2025.1551074

**Published:** 2025-04-10

**Authors:** Dawei Chu, Rui Huang, Jiandang Shi, Ruiqing Xu, Daihao Wei

**Affiliations:** ^1^ First Clinical Medical College of Ningxia Medical University, Yinchuan, Ningxia Hui Autonomous Region, China; ^2^ Department of Orthopedic, General Hospital of Ningxia Medical University, Yinchuan, Ningxia Hui Autonomous Region, China; ^3^ Honghui Hospital, Xi’an Jiaotong University, Xi’an, Shaanxi, China

**Keywords:** osteosarcoma, neutrophil extracellular traps, key genes, therapeutic targets, ATG7, CFH, metastases

## Abstract

**Background:**

Osteosarcoma is the most common primary bone tumor. It has a high rate of early metastasis, and its treatment is one of the most challenging topics in the bone tumor field. Recent studies have shown that neutrophil extracellular traps play an important role in tumor metastasis and may provide new horizons for exploring metastasis in osteosarcoma.

**Methods:**

OS data were downloaded from the TARGET database and Gene Expression Omnibus datasets. Univariate Cox regression was conducted to assess NETRGs. Patients were subsequently categorized into high- and low-risk groups on the basis of risk score values derived from multivariate Cox analysis, and prognostic models were established. The immune infiltration of relevant genes and drug sensitivity of key genes were also analyzed.

**Results:**

A total of 15 NETs-related genes associated with osteosarcoma metastases were identified. Among them, a total of 4 genes were related to prognosis, namely, MAPK1, CFH, ATG7 and DDIT4, and a prognostic model based on these 4 genes was established. The prognosis was worse in the high-risk group, whose areas under the ROC curves (AUCs) were 0.857, 0.779, and 0.689 at 1, 3, and 5 years, respectively. The key genes were subsequently found to be associated with the infiltration of 20 types of immune cells. Finally, the small-molecule drug toxin c 10, an approximately 6700 mw protein, may target key genes. Finally, ATG7 was validated at the histological level by combining the results of the validation group dataset analysis.

**Conclusions:**

A risk model based on 4 NETRDEGs is a reliable prognostic predictor for OS patients, and CFH and ATG7 may serve as a new diagnostic and therapeutic target.

## 1 Introduction

Osteosarcoma is a malignant primary bone tumor that is prevalent in children and adolescents (1–3) and is most common in the distal femur, proximal tibia, and humeral metaphyseal locations (4). It is characterized by the malignant proliferation of prismatic mesenchymal stromal cells that directly produce osteoid or immature bone tissue (5, 6). It is highly malignant with a high rate of early metastasis, and its high propensity for metastasis is a major cause of poor prognosis (7, 8). Simultaneous pulmonary metastases have been reported to occur in approximately 15–20% of patients at the time of initial diagnosis (9–11). Once metastasis occurs in patients with osteosarcoma, the prognosis is extremely poor. Moreover, treatment is of limited importance, with an overall 5-year survival rate of 20%-30% (12, 13), which is much lower than that of patients without metastasis. Osteosarcoma is classified according to the Enneking Surgical Staging System (SSS) into Stage IIA (confined to the anatomical compartment), Stage IIB (breaching bone cortex, fascial tissue, or joint cavity), and Stage III (metastatic) (14, 15). The treatment follows the principle of neoadjuvant chemotherapy → surgery → adjuvant chemotherapy, with first-line chemotherapeutic agents including high-dose methotrexate, doxorubicin, cisplatin, and ifosfamide (16, 17). With the advent of neoadjuvant chemotherapy, limb-salvage surgery has become the primary approach for osteosarcoma with favorable staging. However, the success of surgery depends on achieving safe surgical margins and a favorable chemotherapeutic response. For Stage II A osteosarcoma, limb-salvage surgery is the main treatment (18), but the use of preoperative chemotherapy remains controversial, as chemotherapy insensitivity may lead to tumor progression and worsen prognosis. For Stage II B tumors, preoperative chemotherapy is routinely administered to shrink tumor boundaries, followed by wide resection if vascular or nerve invasion is absent; amputation is required if critical structures are involved (19, 20). For Stage III osteosarcoma, palliative surgery is the primary approach (21). The survival rate of osteosarcoma patients has not improved over the past 30 years, primarily due to the intractability of osteosarcoma metastases in patients (22, 23). Therefore, an in-depth study of the molecules involved in the invasion and metastasis of osteosarcoma cells is urgently needed.

Neutrophil extracellular traps (NETs), NET-like substances released by neutrophils during their immune action against pathogens that can capture and kill microorganisms, were discovered by Volker Brinkmann’s research team in as early as 2004 (24). NETs consist mainly of intracellular DNA, histones and granule proteins, such as myeloperoxidase and neutrophil elastase (25). They are closely associated with the onset and progression of diseases such as infections, sepsis, autoimmune disorders and diabetes (26–28). In recent years, studies involving the interactive functions of neutrophils and the tumor microenvironment have revealed that NETs are involved in the entire invasion–metastasis cascade of a variety of tumors (29, 30). In breast cancer, NET DNA can interact with CCDC25 on tumor cell membranes to activate the ILK-β-Parvin pathway and promote liver metastasis (31). Amyloid-β produced by cancer-associated fibroblasts (CAFs) promotes the generation of NETs by facilitating the production of ROS in neutrophils. NETs promote the hepatic metastasis of pancreatic tumors by enhancing the migration of hepatic stellate cells (32, 33). In addition, NETs contain programmed cell death ligand 1 (PD-L1), which promotes tumor metastasis by binding to programmed cell death protein 1 (PD-1) on the surface of T cells to inhibit T-cell function, leading to T-cell dysfunction and metabolic failure (34). These findings provide new directions for understanding the mechanism of osteosarcoma metastasis and possible future treatments. Therefore, exploring the molecular mechanisms of NET-related genes in the development and metastasis of osteosarcoma is highly important for the early diagnosis and clinical treatment of osteosarcoma patients.

**Figure 1:**
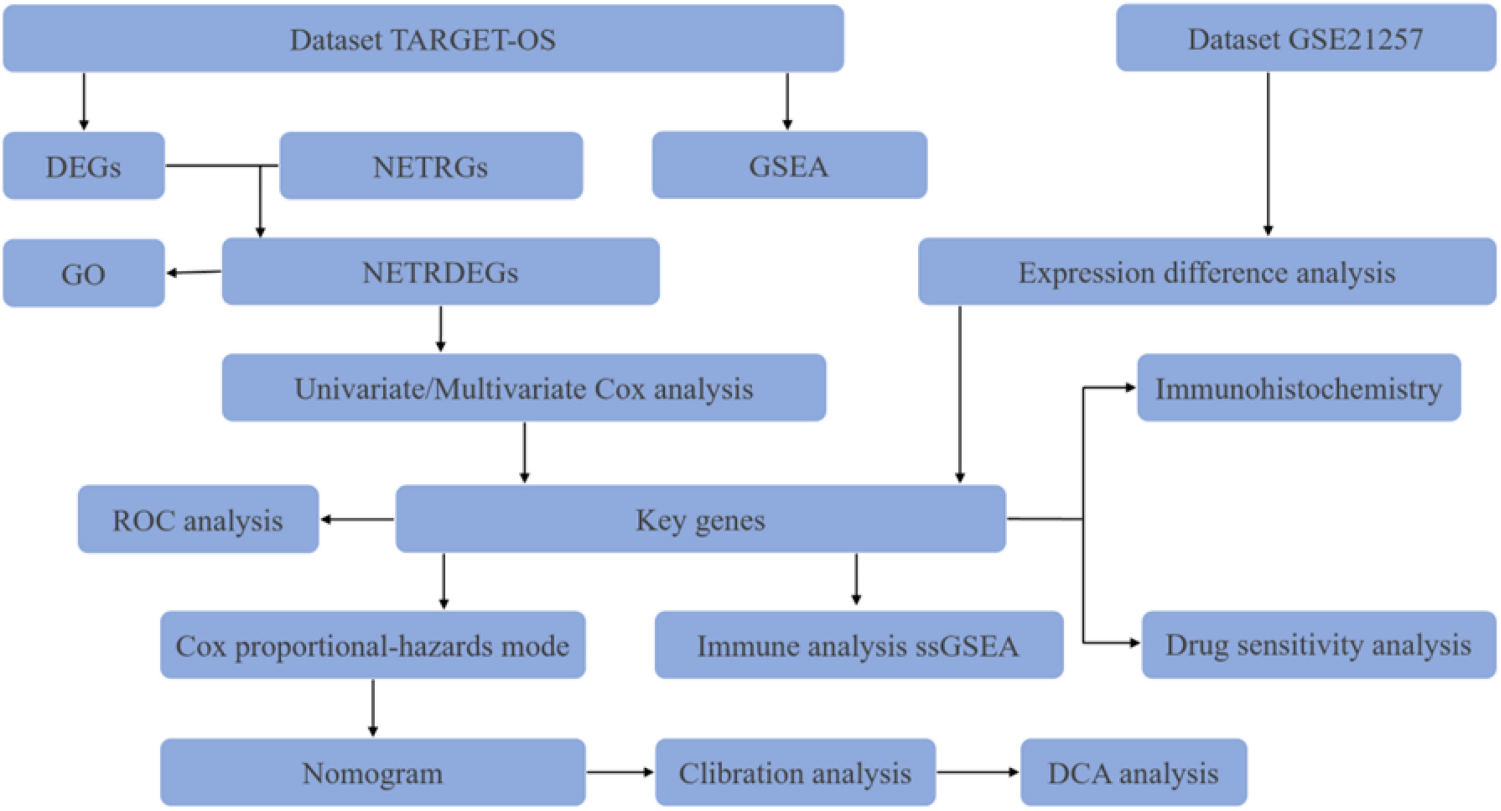
Research flowchart.

Here, we utilized bioinformatics approaches to explore the role of NET-related genes in osteosarcoma metastasis (**Figure 1**). First, we identified MAPK1, CFH, ATG7, and DDIT4 as independent prognostic factors in osteosarcoma patients via Cox regression analysis. In addition, a nomogram graph was established. Analysis of immune infiltration and drug sensitivity of key genes was performed for relevant genes. Finally, ATG7 was validated at the histological level based on the results of the validation group dataset analysis. These results suggest that ATG7 may be a reliable diagnostic and therapeutic target for patients with metastatic osteosarcoma.

## 2 Data and methods

### 2.1 Materials

The expression profile data of TARGET-OS in osteosarcoma patients were downloaded from UCSC Xena (https://xena.ucsc.edu/), and a total of 84 samples were obtained after patient samples with no expression data or survival data were removed. The expression profiling dataset GSE21257 containing metastasis group data was downloaded from the GEO database (https://www.ncbi.nlm.nih.gov/geo/) (**Table 1**). Combined with the Gene Cards database (35) and published literature (36, 37), 258 neutrophil extracellular traps-related genes (NETRGs) were obtained.

**Table 1:** Osteosarcoma dataset information.

	TARGET-OS	GSE21257
Platform	TARGET	GPL10295
Species	*Homo sapiens*	*Homo sapiens*
Tissue	Osteosarcoma tumor tissues	Osteosarcoma tumor tissues
Samples in unmetastases group	63	19
Samples in metastases group	21	34

OS, osteosarcoma; GEO, Gene Expression Omnibus.

### 2.2 Methods

#### 2.2.1 Data collection and processing

The R software “limma” package was used for data correction to ensure the comparability of the data. The samples were divided into 2 groups (the unmetastases group and the metastases group), and the genes whose |log FC| was >0 and *P* value was<0.05 were considered differentially expressed genes (DEGs). The intersections of the DEGs and NETRGs were then plotted as Venn diagrams and differential ordering plots.

#### 2.2.2 GSEA and GO analysis

GSEA was performed via the “cluster Profiler” package and the “c2.cp.v7.2.symbols.gmt” gene set from the Molecular Signatures Database (MSigDB). The parameters were set as follows: the number of seeds was 2020, the number of calculations was 1000, the number of genes contained in each gene set was at least 10, and the maximum number of genes contained was 500. Subsequently, Gene Ontology (GO) functional enrichment analysis was carried out on 15 NETRDEGs.

#### 2.2.3 Construction and validation of the nomogram model

Univariate Cox analysis of NETRDEGs was performed using the “survival” package, and genes with *P*< 0.05 were used as the key genes in our subsequent study. Patients were categorized into high- and low-risk groups based on the median risk score via multivariate regression analysis. A nomogram was constructed using the R software package “rms”, and a decision curve was constructed using the R package “gg DCA” to evaluate the accuracy of the prediction results.


$$\text{RiskScore}=\sum_{\text{i}}\text{Coefficient}\left({\text{gene}}_{\text{i}}\right)\;*\text{ mRNAExpression}\left({\text{gene}}_{\text{i}}\right)$$

In this formula, the coefficient represents the risk factor, and mRNA expression represents the expression value of the gene.

The functional similarity between key genes was calculated using the R package “GO Sem Sim”. The “p ROC” package was used to plot the ROC curves of the key genes.

#### 2.2.4 Immune infiltration and drug sensitivity analysis

Enrichment scores for the level of infiltration of each immune cell type with other stromal cells were calculated using the R software packages “GSVA” and “MCP Counter”. The correlation between immune infiltrating cells was determined via Spearman’s correlation analysis, and *P*< 0.05 was considered statistically significant. The Cell Miner database (https://discover.nci.nih.gov/cell miner/home.do) was searched and based on the expression of the key genes with the drug data in the Cell Miner database. Drug sensitivity analysis of key genes was performed using the “pRRophetic” package.

#### 2.2.5 Immunohistochemical analysis

Osteosarcoma tissue microarrays (Changsha Yaxiang Biotechnologies, Changsha, China) were used for these experiments. The chips were subjected to a dewaxing process and antigen repair, followed by serum blocking to block nonspecific binding. For primary antibody incubation, the samples were rinsed three times with phosphate buffer for 3 min each. Then, the ATG7 antibody (OriGene Technologies, Wuxi, China) was diluted at a ratio of 1:1000, the primary antibody was added dropwise, and the samples were incubated overnight at 4°C in a refrigerator. For secondary antibody incubation, the samples were washed with phosphate buffer three times for 3 min each, horseradish peroxidase-labeled secondary antibody was added dropwise, the samples were incubated at room temperature for 2 h, and the samples were rinsed with phosphate buffer three times for 3 min each. DAB staining solution was added dropwise for coloring, and the color development was observed under a microscope. The samples were quickly washed after coloring. The samples were restrained by incubating them with hematoxylin for 1min, followed by rinsing for 5 min and drying at room temperature before sealing them with a coverslip. The next day, images were obtained with a tissue microarray scanner.

#### 2.2.6 Statistical analysis

R software version 4.1.2 was used for analysis, and R language-related packages (“limma,” “cluster Profiler,” “p ROC,” “rms,” “survival,” “GO Sem Sim,” “gg DCA,” “GSVA,” etc.) were used to process data. Differences in survival were analyzed via the Kaplan–Meier method and are expressed as hazard ratios (HRs) and 95% confidence intervals (CIs). *P*< 0.05 was considered a statistically significant difference. The statistical significance is shown as follows: *P* value< 0.05 (^*^) and *P* value< 0.01 (^**^).

## 3 Results

### 3.1 Standardization of the dataset and screening results of NETRDEGs

**Figure 2:**
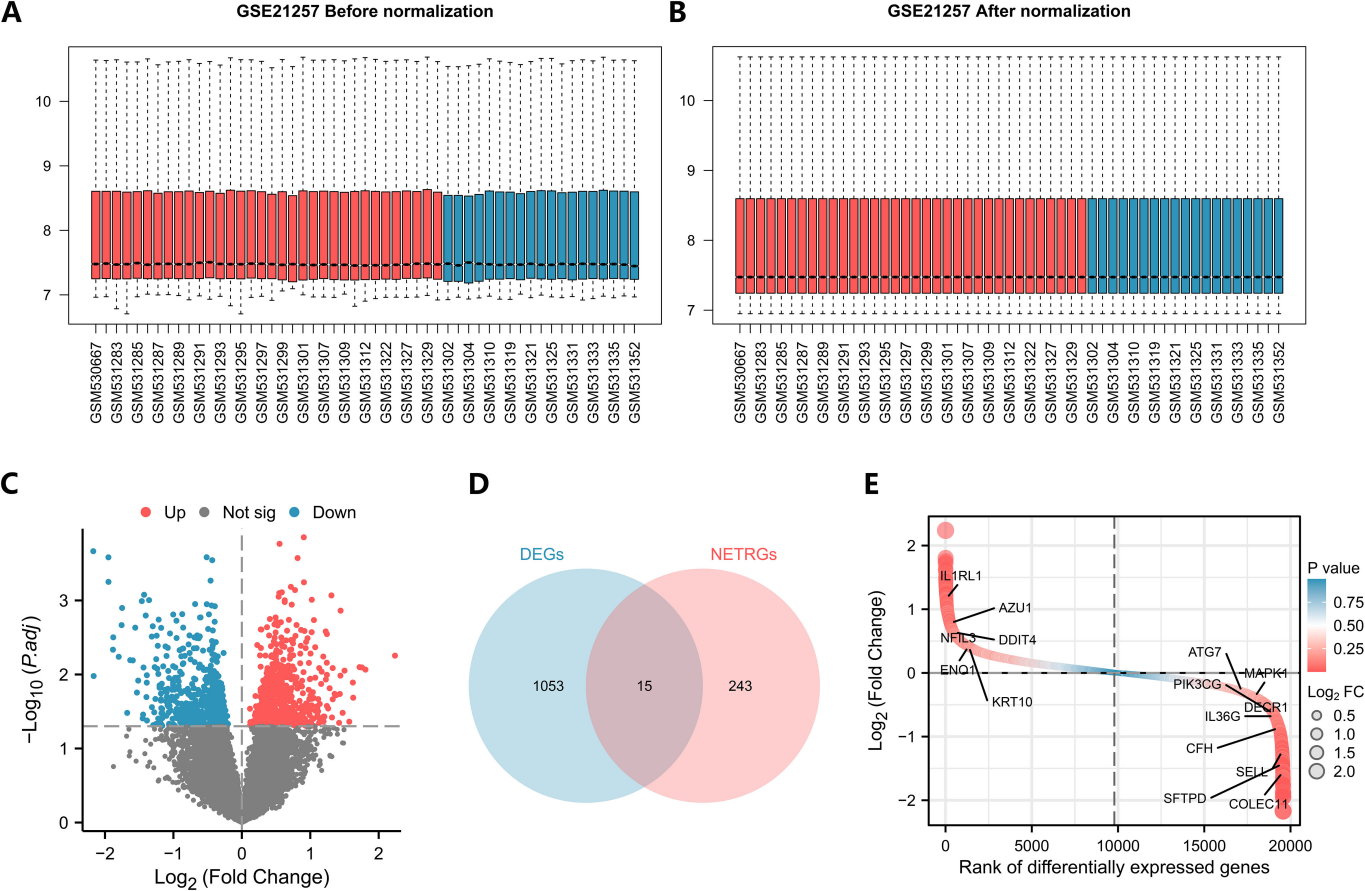
Analysis of TARGET-OS differential genes in the dataset. **(A, B)** Box plots of the GSE21257 dataset before and after correction. (Red represents the metastases group, blue represents the unmetastases group). **(C)** Volcano plots of differentially expressed genes. **(D, E)** Venn diagrams and differential ordering plots of intersecting genes.

The dataset GSE21257 was standardized such that the trend of expression among different samples converged, and a box line plot was drawn for the distribution of data before and after standardization (**Figures 2A, B**). In the dataset, 1068 genes satisfied the threshold of |logFC| > 0 and *P* value< 0.05, and 602 and 466 genes exhibited high and low expression in the metastasis group, respectively (**Figure 2C**). Taking the intersection of all DEGs and NETRGs, a total of 15 NETRDEGs were obtained, including IL1RL1, AZU1, NFIL3, DDIT4, ENO1, KRT10, ATG7, MAPK1, PIK3CG, DECR1, IL36G, CFH, SELL, SFTPD, and COLEC11 (**Figures 2D, E**).

### 3.2 GSEA and GO analysis

**Figure 3:**
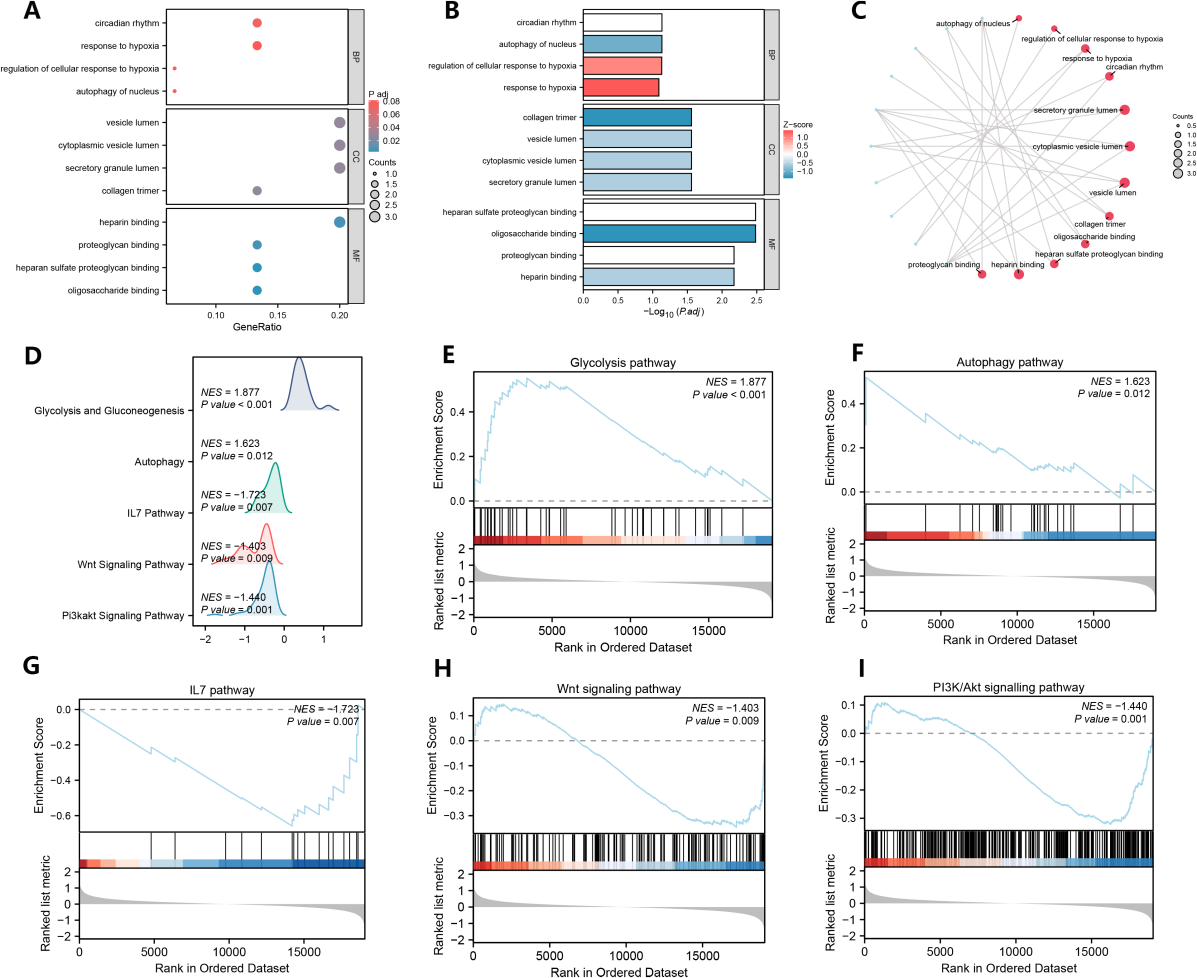
GO analysis of NETRDEGs and GSEA of DEGs. **(A–C)** GO analysis of NETRDEGs. **(A)** bubble chart, **(B)** column chart, **(C)** ring network chart. **(D–I)** Genes associated with the glycolysis pathway **(E)**, the autophagy pathway **(F)**, the IL7 pathway **(G)**, the Wnt signaling pathway **(H)**, the PI3K/Akt signaling pathway **(I)** were significantly enriched.

To analyze the biological functions of the 15 NETRDEGs, we first performed GO analysis of the NETRDEGs (**Figures 3A–C**). These genes were found to be involved in autophagy in the nucleus, the regulation of the cellular response to hypoxia, the response to hypoxia, and the circadian rhythm in biological processes. The cellular component terms were significantly associated with the secretory granule lumen, cytoplasmic vesicle lumen, vesicle lumen, and collagen trimer. The enriched molecular function of the DE-FRGs were as follows: oligosaccharide binding, heparan sulfate proteoglycan binding, heparin binding, and proteoglycan binding. GSEA revealed that the DEGs were significantly enriched in the glycolysis pathway, autophagy pathway, IL7 pathway, Wnt signaling pathway, and PI3K/Akt signaling pathway (**Figures 3D–I**).

### 3.3 Construction of a prognostic model and establishment of a nomogram

**Figure 4:**
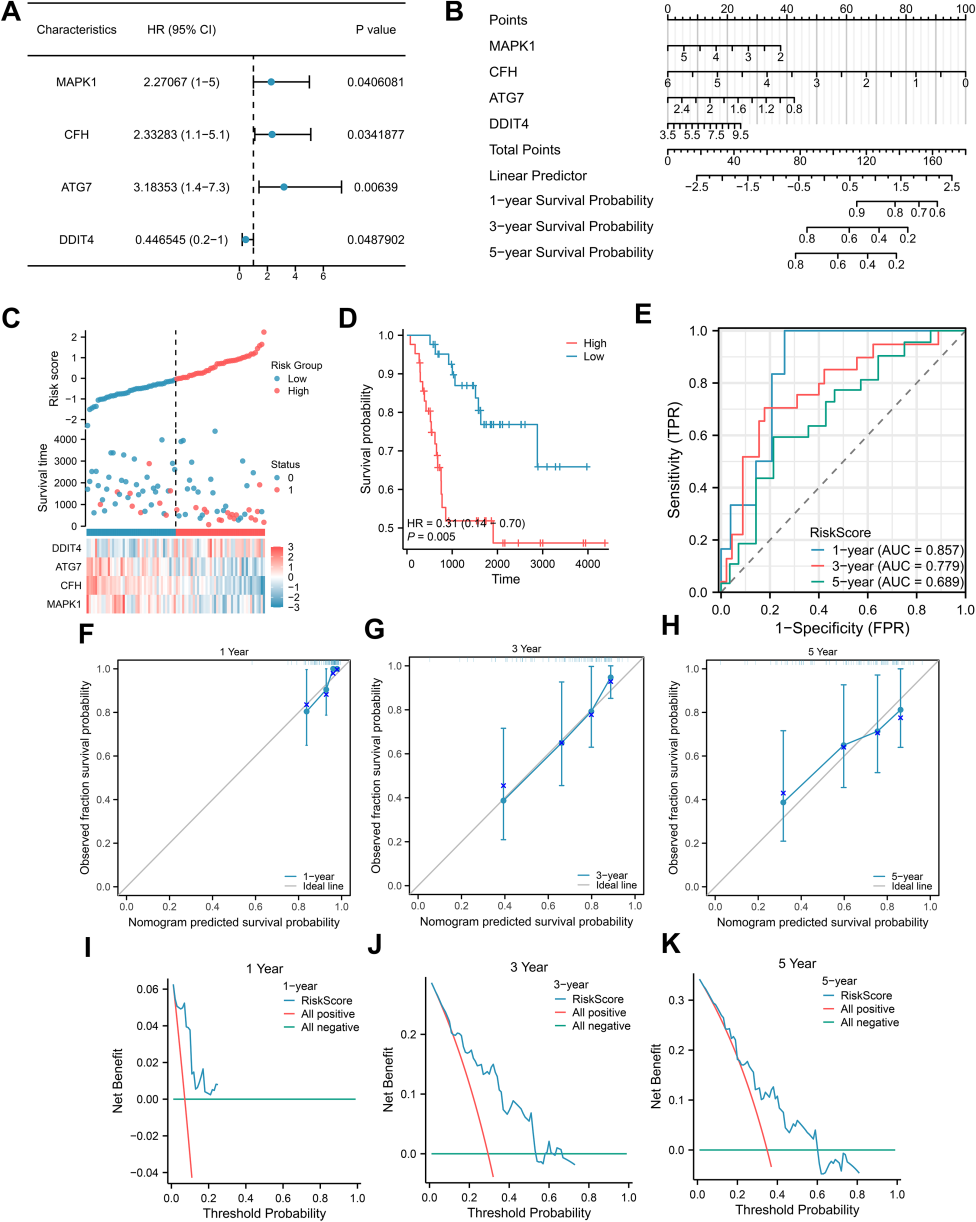
Construction of the Cox regression model. **(A)** Forest plots for univariate Cox regression; **(B)** Nomogram integrating the risk score and clinical characteristics; **(C-E)** Distribution, Kaplan–Meier plot, and time-dependent ROC curve of the risk model. **(F-H)** 1-year, 3-year, and 5-year survival calibration plots of the nomogram. **(I-K)** 1-year, 3-year, and 5-year survival DCA plots of the nomogram.

To obtain a prognostic model for NET-related genes, we screened for NETRGs via univariate Cox analysis in conjunction with survival outcomes and survival times and constructed a forest plot (**Figure 4A**). We then included these key genes in a multivariate Cox analysis to obtain the risk score value and grouped the samples of the dataset into high- and low-risk groups according to the median value of the risk score (cutoff value = -0.050965805) and found that the prognosis was worse in the high-risk group (**Figures 4C, D**). The prognostic model can be expressed as follows: risk score=MAPK1*(−0.350932209)+CFH*(−0.540468911)+ATG7*(−0.765106538)+DDIT4*0.132203877. We then performed a nomogram analysis to determine the prognostic ability of the key genes (**Figure 4B**). The nomogram yields a score for each item, and the total score and corresponding survival rate can be obtained after adding the scores of all the items. The results showed that the utility of the expression of the CFH gene in the model was significantly greater than that of the other genes. Moreover, the AUCs of the 1-, 3- and 5-year ROCs were 0.857, 0.779 and 0.689, respectively (**Figure 4E**). In addition, we performed 1-, 3-, and 5-year prognostic calibration analyses and plotted calibration curves for the prognostic model (**Figures 4F–H**). We found that the model predicted patient survival in general agreement with actual patient survival. We then used decision curve analysis to assess the magnitude of the clinical utility of the constructed models at 1, 3, and 5 years (**Figures 4I–K**), which revealed that the 5-year prognostic model had the best clinical utility.

### 3.4 Prognostic analysis of key genes in the training group

**Figure 5:**
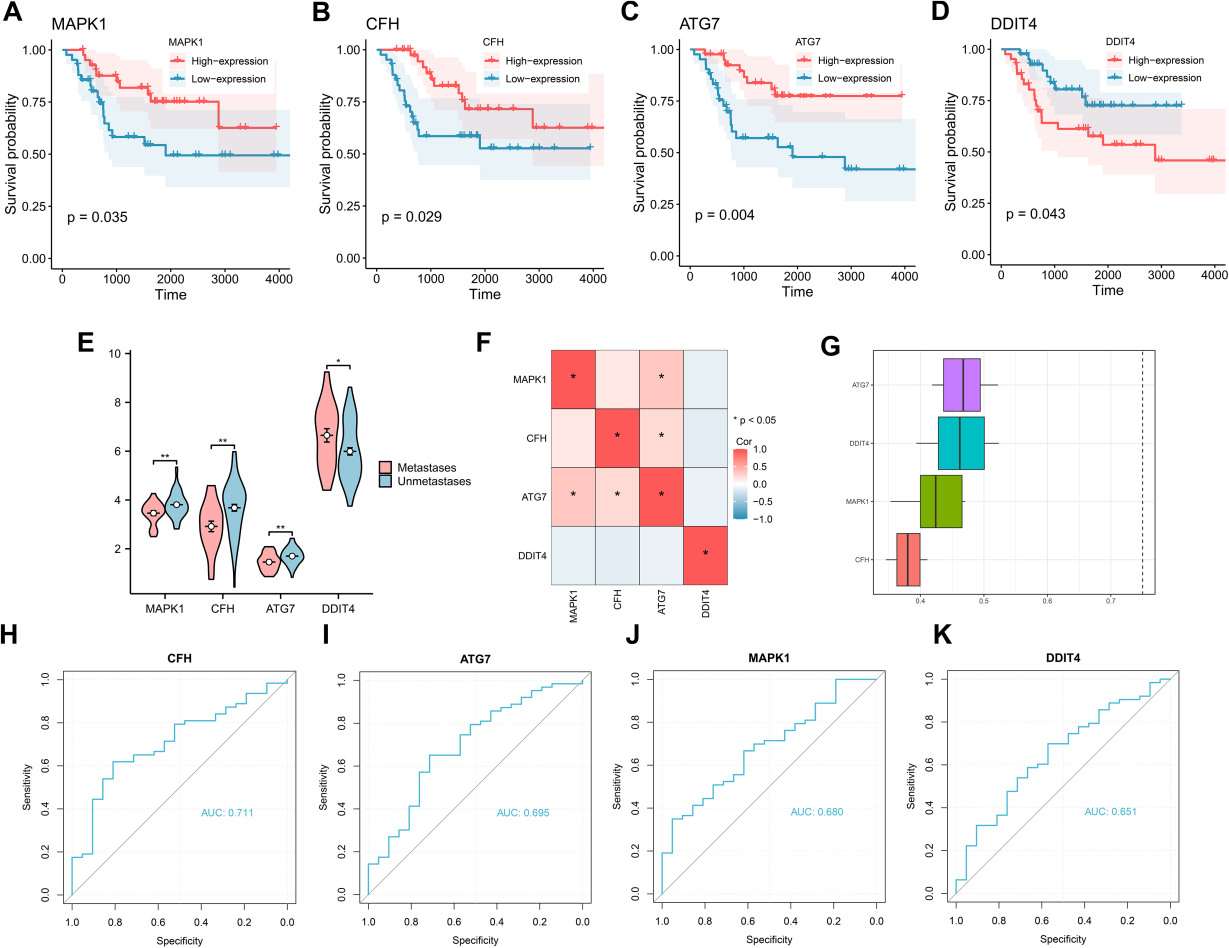
K–M curves and correlation analysis for the TARGET-OS dataset. **(A-D)** K–M curves for prognstic analysis of the genes MAPK1, CFH, ATG7, and DDIT4. **(E)** Group comparison plot of key genes among different subgroups in the TARGET-OS dataset. **(F)** Correlation heatmap of key genes in the TARGET-OS dataset. **(G)** Functional similarity analysis of key genes. **(H-K)** ROC curve analysis of key genes in the TARGET-OS dataset.

To assess the relationships between the four key genes and prognosis, we plotted prognostic Kaplan–Meier survival curves in the TARGET-OS dataset for each of the key genes (**Figures 5A–D**), which revealed that all four genes significantly correlated with survival: MAPK (*P* = 0.035), CFH (*P* = 0.029), ATG7 (*P* = 0.004), and DDIT4 (*P* = 0.043). Group comparison plot of key genes among different subgroups in the training dataset were drawn (**Figure 5E**). In addition, gene correlation analysis was performed based on the complete expression matrix of key genes, and correlation heatmaps were drawn (**Figure 5F**). The results revealed a positive correlation between the genes ATG7 and MAPK1 and between CFH and ATG7. We subsequently performed functional similarity analysis of the key genes and then visualized the results of the functional similarity analysis among the key genes via a box-and-line plot (**Figure 5G**), which revealed that ATG7 was the most similar gene to the other three genes in terms of function. Next, the ROC curves of the key genes were plotted (**Figures 5H–K**). The ROC curves revealed that the differences in the expression of the CFH gene (AUC = 0.711) in the dataset presented comparable accuracy across subgroups.

### 3.5 Analysis of immune cell infiltration

**Figure 6:**
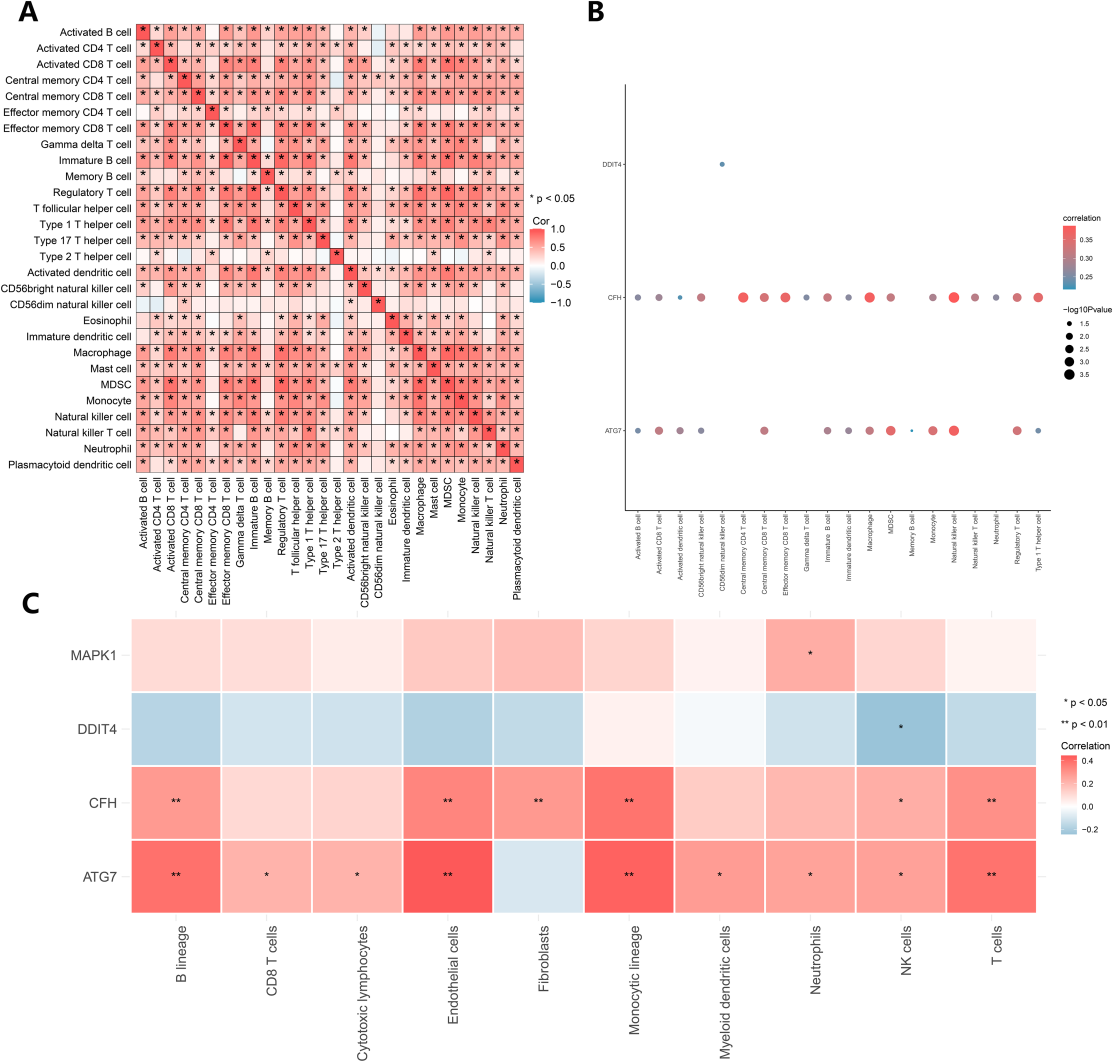
Immune infiltration analysis. **(A)** Correlation analysis of the infiltration abundances of 28 immune cell types calculated via the ssGSEA algorithm. **(B)** The results of the ssGSEA algorithm. **(C)** The results of the MCP Counter algorithm.

To explore immune cell infiltration, the correlation between the infiltration abundance of 28 immune cells was calculated via the ssGSEA algorithm. The results of the correlation heatmap (**Figure 6A**) revealed a positive correlation between the infiltration abundance of immune cells that activated CD8+ T cells and macrophages and between effector memory CD8+ T cells and immature B cells, macrophages and MDSCs. Subsequently, we analyzed the relationships between the key genes and the infiltration abundance of 28 immune cells via the ssGSEA algorithm, and the key genes CFH, ATG7, and DDIT4 were correlated with 20 of these immune cells (**Figure 6B**). Among them, positive correlations were identified between CFH and immune cells, central memory CD4 T cells, natural killer cells, as well as between ATG7 and immune cells and killer cells. To ensure the accuracy of the above algorithm, we also calculated the correlation between key genes and immune cell infiltration abundance via the MCP Counter algorithm (**Figure 6C**), which revealed that the key genes were related to 10 types of immune cells. Among them, positive correlations were identified between ATG7 and endothelial and monocyte lineage cells as well as between CFH and monocytic lineage cells; DDIT4 negatively correlated with NK cells.

### 3.6 Drug sensitivity analysis and immunohistochemical analysis

**Figure 7:**
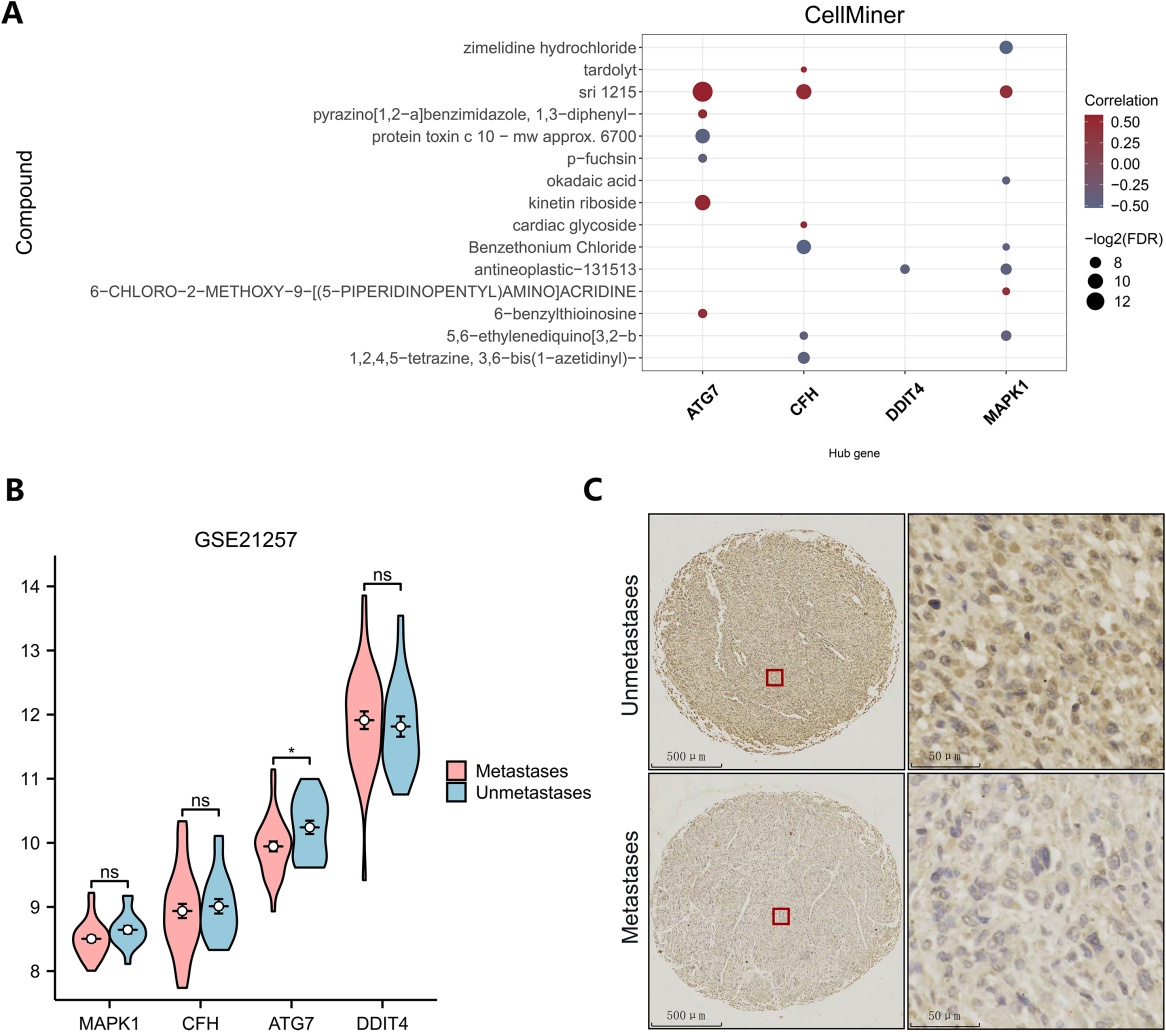
Drug sensitivity analysis and immunohistochemical analysis. **(A)** Drug sensitivity analysis (dark brown represents antagonists, and blue–gray represents agonists). **(B)** Differential expression of key genes between different subgroups in GSE21257. **(C)** Immunohistochemistry of ATG7 in the metastatic and nonmetastatic groups.

To obtain small-molecule drugs that target key genes, we used data from the cancer drug database Cell Miner, including the mRNA expression profiles of key genes and drug activities. Using the pRRophetic algorithm, a ridge regression model was constructed based on the expression and gene expression profiles of the key genes in the TARGET-OS dataset, and the sensitivities of the key genes to common anticancer drugs were predicted by the IC50 values (**Figure 7A**). The results show that key genes can be found in the database Cell Miner for a variety of drugs with interaction relationships. Among them, ATG7, kinetin riboside, MAPK1, and CFH positively correlated with the small molecule sri1215. Negative correlations were identified between ATG7 and the small-molecule drug protein toxin c10-mwapprox.6700, between CFH and benzethonium chloride, and between MAPK1 and zimelidine hydrochloride.

To explore the expression of key genes in different sequencing datasets, the differences in the high- and low-expression key genes among different subgroups in the GEO dataset were analyzed (**Figure 7B**). ATG7 expression was significantly lower in the metastasis group than in the metastasis-free group in the GSE21257 dataset, which was consistent with the analysis of the TARGET dataset. This difference may be a common phenomenon in metastatic patients. However, the expression levels of MAPK1, CFH and DDIT4 did not significantly differ between groups in the GSE21257 dataset, but the trend was consistent with the results in the training set. Low expression of ATG7 is likely a common genetic variant in all patients with osteosarcoma metastases. To assess the potential of ATG7 as a biomarker and therapeutic target for osteosarcoma metastases, we analyzed the expression of ATG7 in tissue microarrays using immunohistochemistry. ATG7 was expressed at low levels at the histological level, which was consistent with the results of our bioinformatics analysis. (**Figure 7C**).

## 4 Discussion

Despite advances in the diagnosis and treatment of osteosarcoma, distant metastasis has become a bottleneck in improving the survival of osteosarcoma patients, which severely restricts their long-term survival (38). In fact, metastasis is a multifactorial and multistep process in which tumor cells undergo three stages: acquisition of *in situ* invasive ability, escape from the immune surveillance system during the circulatory process, and colonization of the premetastatic microenvironment; then, the surviving tumor cells grow in distal organs far from the site of origin, resulting in multiorgan failure (39, 40). Recent studies have demonstrated that the extracellular trap network released by neutrophils during their physiological function is involved in the three stages of the metastatic process to varying degrees, including the establishment of the premetastatic microenvironment, epithelial-to-mesenchymal transition, the colonization of circulating tumor cells, and the growth of tumor cells in micrometastatic lesions (25). However, the role of NETs in the pathogenesis of OS remains poorly understood, prompting us to explore the possibility of using NET-related genes as OS biomarkers.

Here, we functionally analyzed osteosarcoma NET-related genes via bioinformatics methods. A new prognostic risk model associated with osteosarcoma NETs was identified, and the correlations of the associated genes with the immune microenvironment and small-molecule drugs were also analyzed. Previous studies have indicated that NETs-related genes play a significant role in osteosarcoma metastasis and immune cell infiltration, with notable differences in key immune components, such as natural killer T (NKT) cells and CD4 T cells, observed between metastatic and non-metastatic groups (41, 42). Importantly, in their study, compared to the low NETscore group, the high NETscore group exhibited significantly lower scores in immune function-related aspects, including immune checkpoints. Using different research methods, we further confirmed that CFH, functioning similarly to an immune checkpoint, plays a crucial role in immune regulation. CFH may help tumor cells evade immune surveillance, promoting metastasis. What’s more, we firstly found that autophagy pathways were significantly enriched in the metastatic group. ATG7, as an important member of the autophagy family, showed significant differential expression in our study. This indicates that ATG7 plays a crucial role in osteosarcoma autophagy, underscoring its importance for further research. Additionally, we identified small-molecule drug agonists or inhibitors targeting key genes, providing potential therapeutic avenues for further exploration.

Recent developments in the field of immunotherapy have facilitated in-depth studies of the osteosarcoma tumor microenvironment, where immune cells within the TME play a key role in osteosarcoma genesis and influence the therapeutic response and clinical outcomes (43). Previous studies have demonstrated that many neutrophils in the tumor microenvironment are affected by CXCR1- and CXCR2-activating ligands produced by tumor cells, which induce the production of NETs to shield immune cells (CD8+ T cells and NK cells) from exposure to tumor cells, thereby preventing tumor cells from being killed by immune cells and facilitating tumor metastasis (27, 44). Therefore, to further clarify the driving role of immune cells in osteosarcoma metastasis, we explored the infiltration of NET-related genes by various immune cells. We found that these four genes were significantly associated with the infiltration of 20 types of immune cells, including T cells and NK cells (45). These findings confirm that key genes play important roles in tumor immunity and provide new ideas for osteosarcoma immunotherapy. Most importantly, our study found a statistically significant difference in CFH between the two groups. As a crucial regulatory protein of the complement system, although CFH is not a typical immune checkpoint molecule (such as PD-1 or CTLA-4), it plays a “checkpoint-like” role in immune regulation, primarily by modulating the activity of the complement system to maintain immune homeostasis and prevent excessive immune responses and tissue damage (46). As is well-known, immune checkpoints such as PD-1 act as “switches” of the immune system (47). Their dysregulation compromises the body’s immune surveillance of tumor cells and plays a critical role in tumor metastasis. Specifically, as an inhibitor of the complement system, CFH can prevent inflammatory responses and tissue damage caused by overactivation of the complement system, thereby inhibiting complement-mediated autoimmune reactions and maintaining the balance of the immune system. Furthermore, CFH can exert antimetastatic effects by inhibiting excessive angiogenesis in tumor tissues (48, 49). In our study, we observed reduced expression of CFH in the metastatic group, and we believe that CFH plays a significant role in the imbalance of the complement and immune systems in osteosarcoma. This finding is important for further research and will be a key focus of our next steps.

MAPK1, also known as extracellular signal–regulated kinase (ERK2), is an important component of the MAP kinase signal transduction pathway. It plays an important role in regulating cell proliferation, differentiation, apoptosis, migration and other activities (50). Studies have shown that aberrant activation of ERK2 in the MAPK pathway is an important cause of a variety of cancers, such as oral cancer (51) and hepatocellular carcinoma (52), in which the hyperactivation of ERK2 can be detected. In addition, a study revealed that this protein, which is a moonlighting protein, also has a transcriptional repressive effect independent of kinase activity. Specifically, IFNγ signaling leads to ERK overactivation in melanoma cells, followed by the generation of an overstress response that leads to cell death. Moreover, the overexpression of either ERK1 or ERK2 leads to cell death in human melanoma cell lines (53). In our study, MAPK1 was expressed at low levels in the training set, but this difference was not significant in the validation set GSE21257. We speculate that the reason for this difference may be related to differences in the site of metastasis and the heterogeneity of the tumor, resulting in different molecular biological alterations, however, this hypothesis needs to be verified in larger studies.

The relationship between cancer and autophagy is complex and is characterized by the fact that the pro– and anticancer properties of autophagy are mutually transformative under specific circumstances (54–57). As an important autophagy effector enzyme, ATG7 can regulate immunity, cell death, and protein secretion together with other autophagy–associated proteins and independently regulate the cell cycle and apoptosis (58). ATG7 multifunctionality is reportedly associated with oncogenic or pro–oncogenic properties in different tumors. Studies have reported that ATG7 deficiency in mice leads to hepatocellular carcinoma by activating the Yap metabolic pathway (59). In another study, elevated ATG7 expression was associated with bladder cancer (60) and lung cancer (61), and high levels of ATG7 expression were associated with poor prognosis in breast cancer patients (62). Other studies have shown that whether ATG7 promotes or suppresses tumors also seems to depend on the status of the tumor suppressor P53 (63, 64). Our findings suggest that ATG7 may suppress metastasis, and its association with the status of P53 has not been reported in the field of osteosarcoma and warrants further investigation. Although the complex link between ATG7 and osteosarcoma remains puzzling, alterations in autophagy are increasingly associated with tumors, and targeting and regulating ATG7 may constitute a promising therapeutic approach.

DNA damage–inducible transcript 4 (DDIT4) is a tumor–associated protein that is highly expressed under stress conditions, such as chemotherapy, heat shock, energy depletion, hypoxia and DNA damage. It is involved not only in tumor survival, antitumor resistance and antiapoptotic processes but also in tumor metastatic behaviors, such as proliferation and invasion (65, 66). Recent analyses of DDIT4 in several cancer types have shown that high expression of this gene is associated with poor prognosis in several hematological and solid tumors, such as acute myeloid leukemia (67), breast cancer (68) and lung cancer (69). In terms of mechanism, DDIT4 is involved in the mTORC1, p53, HIF, autophagy and oxygen sensing signaling pathways through intermolecular interactions with multiple pathway proteins. It is directly involved in the activation of several important pathways and has a driving role in tumor progression and metastasis (70, 71). This finding is consistent with our findings and can be used as a new therapeutic strategy to provide a research basis.

However, our study is subject to several shortcomings. First, the data used in our study were not our own but were obtained from public databases, and whether the sequencing data in the databases can reflect the genetic alterations in all patients remains to be demonstrated. Second, due to the lack of clinical samples from osteosarcoma patients, the key genes could not be quantitatively analyzed by RT–qPCR and WB experiments. Third, the specific mechanisms of ATG7 and CFH with respect to the OS autophagy and immune microenvironment have not been further investigated. More prospective studies are needed if the value of NETs in OS metastasis is to be further confirmed.

## 5 Conclusion

In conclusion, we developed a prognostic model based on four NETRDEGs, namely, MAPK1, CFH, ATG7 and DDIT4. ROC curves and nomogram plots were used to assess the accuracy of the model, which demonstrated that our prognostic model could reliably predict OS outcome. Furthermore, our study revealed that NETRDEGs can influence immune cells within the tumor microenvironment, and CFH may play a “checkpoint–like” role in the immune regulation of osteosarcoma. More importantly, ATG7 plays a significant role in osteosarcoma autophagy, providing new clues for exploring immunotherapeutic approaches for osteosarcoma patients.

These findings may lead to new therapeutic targets for the diagnosis and treatment of metastasis in OS patients, and more relevant studies are needed to further validate the link between CFH, ATG7 and osteosarcoma metastases. This study provides a basis for exploring the molecular mechanisms, diagnosis and treatment of osteosarcoma metastases.

## Data Availability

The data presented in the study are deposited in the GEO data base (https://www.ncbi.nlm.nih.gov/geo/) and UCSC Xena (https://xena.ucsc.edu/) repository, accession number GSE 21257.
